# Editorial: GenAI in healthcare: technologies, applications and evaluation

**DOI:** 10.3389/frai.2026.1878137

**Published:** 2026-06-17

**Authors:** Tim Hulsen, Mark C. Scheper, Steffen C. Pauws

**Affiliations:** 1 Data Science and AI Engineering, Philips, Eindhoven, Netherlands; 2 AI and Data Supported Healthcare: Data-Science Unit, Research Center Innovations in Care, Rotterdam University of Applied Sciences, Rotterdam, Netherlands; 3 Medical Delta Living Lab Data Supported Healthcare and Innovation, Medical Delta, Delft, Netherlands; 4 Allied Health Professions, Faculty of Medicine and Science, Macquarrie University, Sydney, NSW, Australia; 5 Electric Engineering/Signal Processing Systems Biomedical Diagnostic (BM/d) Lab, Eindhoven University of Technology, Eindhoven, Netherlands; 6 Department Communication and Cognition, Tilburg University, Tilburg, Netherlands

**Keywords:** artificial intelligence, GenAI, generative adversarial network (GAN), healthcare, large language model (LLM), retrieval-augmented generation (RAG)

## Introduction

Generative Artificial Intelligence (GenAI) is a relatively new type of Artificial Intelligence (AI), capable of generating text, images, videos, and other data or content. GenAI has captured massive global attention thanks to the remarkable success of tools like ChatGPT, Google Gemini, Claude, and DeepSeek. It is already having a profound and transformative impact in entertainment industries such as gaming, movies, literature and music and all other key industries such as finance, marketing, retail and software. In healthcare, GenAI is being applied as well to automate administration, assist in clinical decision-making, and personalize patient care. Seemingly permitted under strict regulatory conditions, it can help healthcare providers by writing summaries of radiology and pathology reports or electronic health records (EHRs), creating patient-facing chatbots empowered with the latest medical knowledge, creating synthetic data based on privacy-sensitive real data, and much more.

In this Research Topic, we have created an overview of applications and evaluations of GenAI in healthcare, using, for example, large language/vision models (LLMs/LVMs), generative adversarial networks (GAN) and retrieval-augmented generation (RAG). We present several examples of successful implementations and evaluations of GenAI in healthcare, and its way toward deployment, but, since this area of research is developing fast, we also look at its possibilities and necessities in the (near) future. As healthcare has a high stake in patient safety, privacy and diagnostic accuracy, some manuscripts also discuss risks and pitfalls around GenAI in healthcare, and the possible solutions for these issues.

The fourteen articles included in this Research Topic have been divided into four sections:

- Clinical deployment, decision support & real-world evaluation
- Education, assessment & quality assurance
- Documentation, data extraction, synthetic records & platform engineering
- Methods, prompting, measurement & specialized NLP applications

## Articles

### Clinical deployment, decision support, and real-world evaluation

The following four papers describe the practical use of LLMs/GenAI in clinical workflows, decision support, and patient-facing care. Papers in this group evaluate concordance with clinicians, usability in digital health interfaces, and governance/quality frameworks needed for safe deployment in real-world clinical settings.

Workum et al. explores the potential of LLMs in healthcare, highlighting their applications in administrative tasks and clinical decision support (CDS). LLMs have demonstrated superior performance in diagnosing complex cases compared to human clinicians and efficiency in summarizing medical records. The ACUTE mnemonic is introduced to evaluate LLM outputs based on Accuracy, Consistency, semantically Unaltered outputs, Traceability, and Ethical considerations, ensuring safe implementation. While CDS applications require strict regulatory compliance, non-CDS uses lack robust oversight. The study emphasizes the importance of structured evaluation, healthcare-specific adaptations, and governance to maximize LLM benefits while minimizing risks in clinical and administrative settings.

Déniz et al. evaluates the concordance between Scholar GPT (a GPT-4-based LLM) and thoracic surgeons' decisions during 81 outpatient consultations at a tertiary hospital. Using a 0–5, the mean concordance score was 3.67, with high agreement (scores 4–5) in 56.8% of cases, particularly for oncological diagnoses like mediastinal and pleural tumors. Lower concordance occurred in complex conditions such as metastatic lung disease and thoracic outlet syndrome. No significant differences were found across visit types or modalities. LLMs show promise in guideline-driven scenarios, but limitations include variability in complex cases, lack of interpretability, and safety concerns, underscoring the need for human oversight.

Imrie et al. explores the integration of LLMs into digital health interfaces, emphasizing their potential to enhance healthcare delivery by improving usability and trust. It highlights the QRisk3 model for cardiovascular disease risk assessment, which incorporates new variables like corticosteroid use due to its association with increased cardiovascular risk. The study demonstrates the effectiveness of LLMs in providing nuanced, real-time clinical insights, outperforming standalone models like GPT-4 in complex queries. While LLM-based systems show promise in streamlining patient management and reducing clinician burnout, challenges such as regulatory compliance, data privacy, and computational costs remain critical considerations.

Skjervold et al. explores GPT-4o's potential in diabetes care. The study compared GPT-4o responses to those from healthcare professionals across 113 patient questions, rated by 273 participants (patients and clinicians) on knowledge, helpfulness, and empathy. GPT-4o was preferred in 46.7% of evaluations vs. 23.3% for humans, with small but statistically significant advantages, especially among participants with lower education. Ratings correlated strongly across quality dimensions, and GPT-4o scored higher for longer questions. While perceived quality was higher, clinical accuracy was not assessed. Authors stress ethical, safety, and oversight considerations before clinical deployment.

### Education, assessment, and quality assurance

These four papers describe applications of GenAI for teaching, assessment, and patient education. This group covers AI-generated exam content, medical student/resident support, and use of conversational models to produce understandable patient information—emphasizing learning effectiveness, clarity, and the need for human validation.

Chokkakula et al. studies the transformative role of ChatGPT in medical education, emphasizing its ability to provide personalized learning experiences, simulate clinical scenarios, and enhance student engagement. By leveraging real-time updates, research papers, and large datasets, ChatGPT offers adaptive, interactive, and evidence-based learning, surpassing traditional methods limited to fixed syllabi and standardized assessments. It aids in clinical decision-making, research proposal generation, and diagnostic accuracy, though its recommendations require validation and human oversight.

Rivera-Rosas et al. explores the use of ChatGPT-3.5 as an assessment tool for medical residents in Mexico, focusing on its ability to generate multiple-choice question exams for topics like Pertussis and Rocky Mountain Spotted Fever (RMSF). Assessments were administered to 35 residents across anaesthesiology, emergency medicine, and internal medicine specialties, with average scores of 8.46 and 8.29 for the respective exams. Residents found the exams well-written, clear, and coherent, though 25.71% reported some confusion in the language.

Lin et al. examines the effectiveness of Google and ChatGPT compared to doctors in educating parents about congenital cataracts. Using two question banks, the study evaluates the correctness, completeness, readability, helpfulness, and safety, finding that ChatGPT excels in readability and completeness but requires human oversight due to risks like hallucinations. Readability enhancements significantly improved metrics such as Flesch Reading Ease and reduced the percentage of difficult words, aligning better with recommended standards for patient education. This paper highlights the potential of AI tools as supplementary resources for healthcare education while emphasizing the importance of ethical use and expert validation.

Zhu et al. evaluates the performance of three AI language models (Deepseek V3, Doubao, and Kimi1.5) in answering 16 chronic non-bacterial osteitis (CNO)-related questions based on expert consensus. Doubao consistently provided the fastest responses and the longest answers, while Deepseek V3 took the most time to respond. Kimi1.5 offered the shortest answers but achieved the highest accuracy scores across multiple trials. Independent evaluations by two reviewers showed consistent scoring, though minor discrepancies arose due to subjective judgment. This underscores the value of AI in healthcare education for rare conditions and highlights the need for consulting multiple information sources for comprehensive health guidance.

### Documentation, data extraction, synthetic records, and platform engineering

This section contains four papers that are about automating and engineering data workflows—finetuning/localizing models for clinic-specific document generation, extracting structured data from unstructured clinical notes, creating synthetic EHRs, and building integrated platforms (RAG/Text-to-SQL/agents) to support regulatory or research tasks.

Hou et al. explores the fine-tuning of the LLaMA-3 model for automated physician letter generation in radiation oncology, addressing the time-intensive nature of manual letter writing. Using a dataset of 14,479 letters, the model was trained with QLoRA on limited computational resources, ensuring privacy-preserving practices. The model demonstrated adaptability to clinic-specific styles and physician preferences, such as differentiating between “planned treatment” and “recommended treatment.” Evaluated across 10 cases, it showed decent performance but variability in quality. The authors highlight the potential of localized LLMs to streamline clinical workflows while emphasizing the need for further refinement and testing.

Poser et al. discusses a semi-automated approach to extract structured data from unstructured clinical reports of Multiple Sclerosis (MS) patients using LLMs. By combining outputs from three different LLMs (OpenAI, Anthropic, and Google) into a consensus model, the study demonstrates improved accuracy and reliability compared to manual evaluations. The process involved analyzing 30 anonymized outpatient reports, refining prompts iteratively, and evaluating 19 clinical variables of varying complexity. Results showed that LLMs can match or even surpass medical professionals in certain scenarios, highlighting their potential to streamline clinical data extraction while reducing time, cost, and bias.

van Velzen et al. explores the generation and evaluation of synthetic Dutch-language electronic health records (EHRs) for patients with lower back pain, using GenAI-assisted methods. It describes the original dataset of 13 real-world EHRs, which included structured and unstructured text, and the development of Python scripts to extract linguistic and informational parameters. Synthetic EHRs were assessed for structural fidelity, linguistic quality, and informational content using a benchmark framework with metrics like word count, document length, and entropy. While synthetic records mirrored real EHRs in style and phrasing, they were shorter (approximately 30%) and less detailed.

Vieira-Vieira et al. introduces PRINCE (PReclinical INformation CEnter), a cloud-hosted data integration platform developed by Bayer AG in collaboration with Thoughtworks, aimed at enhancing drug development while reducing costs in a complex regulatory environment. PRINCE utilizes a multi-agent architecture powered by LLMs and advanced data retrieval techniques like Retrieval-Augmented Generation (RAG) and Text-to-SQL to integrate decades of structured and unstructured preclinical safety study reports. Evolving from a basic keyword search tool to a sophisticated research assistant, PRINCE now answers complex queries and drafts regulatory-critical documents.

### Methods, prompting, measurement, and specialized NLP applications

This final section contains two papers about methodological advances and specialized evaluation techniques for applying LLMs in healthcare, including prompt engineering to improve statistical reasoning, and using PLMs for quantitative measurement in clinical research.

Vilakati explores the use of prompt engineering to enhance statistical reasoning with LLMs in medical research. It compares various prompting strategies, such as zero-shot, few-shot, chain-of-thought, and hybrid approaches, highlighting their advantages and limitations. A structured framework is proposed for inferential tasks, including assumption testing, model selection, and prompt formulation. Evaluation of LLM outputs, using tools like Microsoft Copilot and human review, focused on criteria such as assumption checking, test selection, and interpretive quality. Results demonstrate that context-rich and structured prompts significantly improve statistical accuracy and reproducibility.

Cong and Lee investigates the use of pre-trained language models (PLMs) to track language recovery in individuals with aphasia following structural priming treatment. Using PLM-derived surprisal scores, the authors quantify syntactic alignment and treatment-induced changes. Results show significant surprisal reductions post-training, especially in participants with severe impairments, indicating improved sentence production. PLMs also demonstrated strong performance in automated assessment tasks, achieving F1 scores up to 0.97 for correctness and 0.85 for error-type classification via few-shot prompting. Findings suggest PLMs offer interpretable, scalable tools for monitoring rehabilitation, enabling personalized therapy and advancing AI integration in clinical language recovery.

## Discussion

Since the public release of OpenAI's ChatGPT in November 2022, the use of GenAI has exploded as well as its further innovation. The current pace of innovation exceeds the adaptive capabilities of most industries, with healthcare not excluded. As highlighted in this special issue, GenAI has already made a lasting impact in healthcare spanning from privacy enhancing technologies through data-synthetisation to LLM-based systems for textual content analysis. While these examples showcase the potential of Gen-AI for healthcare, major challenges remain that not only hurdle further innovation but also severely constrain the clinical value of these applications.

First, it is important to realize that most (generative) AI technologies are developed for the medical professions, where data are typically highly standardized and specialized for medical imaging and medical devices. However, about 80% of healthcare delivery happens outside these professions, but within nursing and allied healthcare professions instead. Nurses, physical, occupational, speech therapists and social workers all play a crucial role in healthcare, yet AI innovation is limited for these professions. This limitation is largely due to the nature, quality and availability of data. About 90% of healthcare data is unstructured (e.g., free text) or formatted across various classification standards. Less than 2% of healthcare data is readily reuseable or accessible. In addition, the commercial incentive to develop AI for these professions is constrained by regulatory requirements, including the Medical Device Regulation (MDR) and the AI Act.

Second, although AI is widely expected to transform care delivery, levels of digital and AI literacy remain disturbingly low among healthcare professionals and patients. AI is not considered amongst the basic competences of a healthcare professional, with only little awareness about the minimum level of AI literacy required for its responsible use. Given the fast pace in AI innovation, skills, knowledge and awareness of its use are often lagging behind. To move forward responsibly, the healthcare sector must do more than developing or supporting AI innovation. It also must establish theoretical frameworks for grounding the use of AI in fundamental processes like clinical reasoning, shared decision making and tailored care.

Lastly and perhaps most important is the patient perspective. Patients have the right to accept or refuse the use of AI in their treatment. Moreover, they also have the right to understand AI in their treatment. This means that AI literacy cannot be limited to healthcare professionals only but must be extended to everyone irrespective of socio-economic background. GenAI is rapidly reshaping the world as we know it, meaningful and responsible AI innovation in healthcare will need fundamental reforms in the knowledge, skills and competences of healthcare professionals. Only then we can achieve innovations in healthcare that are sustainable, safe and meaningful for a better future.

